# Approaches to Integrating Metabolomics and Multi-Omics Data: A Primer

**DOI:** 10.3390/metabo11030184

**Published:** 2021-03-21

**Authors:** Takoua Jendoubi

**Affiliations:** Department of Statistical Science, University College London, London WC1E 6BT, UK; t.jendoubi@ucl.ac.uk

**Keywords:** data integration, multi-omics, integration strategies, genomics

## Abstract

Metabolomics deals with multiple and complex chemical reactions within living organisms and how these are influenced by external or internal perturbations. It lies at the heart of omics profiling technologies not only as the underlying biochemical layer that reflects information expressed by the genome, the transcriptome and the proteome, but also as the closest layer to the phenome. The combination of metabolomics data with the information available from genomics, transcriptomics, and proteomics offers unprecedented possibilities to enhance current understanding of biological functions, elucidate their underlying mechanisms and uncover hidden associations between omics variables. As a result, a vast array of computational tools have been developed to assist with integrative analysis of metabolomics data with different omics. Here, we review and propose five criteria—hypothesis, data types, strategies, study design and study focus— to classify statistical multi-omics data integration approaches into state-of-the-art classes under which all existing statistical methods fall. The purpose of this review is to look at various aspects that lead the choice of the statistical integrative analysis pipeline in terms of the different classes. We will draw particular attention to metabolomics and genomics data to assist those new to this field in the choice of the integrative analysis pipeline.

## 1. Introduction

Biological processes and functions are the result of multiple interactions between tens of thousands of molecules and are inherently complex. In the last 30 years, the parallel acquisition of high-throughput multi-omics datasets from the metabolome, genome, epigenome, proteome, and transcriptome has seen a tremendous boost. As a result, integrative analysis methods for multi-omics data are emerging and gaining popularity among researchers. Integrative analysis consists of the combination of the information available from multi-omics data to provide an enhanced readout of cellular processes and molecular programmes in multiple fields encompassing plant biology [1], animal science [2], toxicology [3,4], molecular epidemiology [5,6], and complex diseases [7,8].

The genome, metabolome, proteome, and transcriptome form different layers of the so-called omics cascade, each of which characterizes a biosystem or an organism at different biomolecular levels [9]. The terms “multi-omics” or “cross-omics” are often used to reflect the heterogeneity of biomolecular profiles and complexity of omics layers they try to measure. Integrating different omics profiles helps extract insightful information and appreciate more comprehensive snapshots of biological systems and molecular processes. Integrative analysis has been applied to associate omics entities to a phenotype of interest e.g., cardiovascular disease [10], cancer [11], or a given treatment or intervention [12]. Other applications of multi-omics analysis include cross-omics biomarker discovery [13,14,15], patient stratification [16,17], and functional analysis [18,19].

In fact, the need for data integration is naturally explained by the complex processes involving e.g., genetic variants, microorganisms, post-translational modifications, metabolic processes, and the biological interrelationship between the different types of omics entities —the collection of which determines the biological state of a living organism [20,21]. In the early 2000s, multi-omic studies of genomic and metabolomic data have identified several alleles that explain a significant proportion of the variation in the metabolic profile [15,22,23]. Similarly, large population studies have linked sequence variations to changes in lipid profiles [24]. Conversely, metabolites can be involved in consequential reactions reaching as deep as cell building blocks [25]. For instance, metabolic fingerprints can help pinpoint genes that affect metabolism and provide functional insight by mapping back to the function of the gene [26]. Similarly, metabolites contribute to reinforcing gene annotations by identifying downstream targets from a specific gene [27].

An arsenal of mathematical and computational techniques was developed to achieve integrative analyses ranging from least squares-based models [28] to Bayesian models [29] and deep learning models [30,31]. In the era of high-throughput data, it became necessary to look into the fundamentals of integrating multi-omics data to provide early career researchers with a guidance on considerations that should be regarded when developing multi-omics data integration methods. In this review, we focus on principles of integrative analysis using five criteria, hypothesis, data types, strategies, study design, and study focus to assist early career researchers in the choice of options that integrative analysis offers. Based on these criteria, we also examine types of statistical data integration under which all existing methods fall. Table 1 provides a review of this primer on data integration and summarizes the different categories that we discuss later. We acknowledge that there are various surveys on statistical multi-omics integrative methods in the literature [28,32,33,34,35,36,37]; however, these often focus on one specific aspect of data integration. By contrast, our review covers more comprehensive discussions on a higher level about the heuristics of data integration and the considerations relevant to the integrative analysis process. Table 2 focuses on two case study examples cited in Table 1. The table depicts considerations that authors addressed to carry out appropriate multi-omics experiments and integrative analysis in both examples. We review these considerations in further detail in this work. In the following section, we discuss the challenges that arise when combining multi-omics data. In the next sections, we shall examine data integration methodologies according to the five criteria: study design, hypothesis, data types, strategies and study focus respectively.

## 2. Challenges in Metabolomics and Multi-Omics Data Integration

When dealing with metabolomics data for integrative analysis, multiple challenges arise and these are in some cases shared with the other omics. On a first instance, omics are not fully characterized. Profiling technologies in metabolomics are subject to an incredibly complex chemical heterogeneity where metabolites are typically not easily identified. The metabolome, in fact, is characterized by a high diversity comprising thousands to hundreds of thousands of chemicals [55]. As a consequence, unknown metabolic entities may be not only unidentified, but may also represent chemicals that have not been reported in the literature. On the other hand, genomic variables are not usually fully characterized by the profiling technology and require an annotation step. Gene annotation is subject to two major bottlenecks: Identifying elements on the genome (Gene finding) and adding biological information to these elements (Gene function). Uncovering the function of genes is critical to understanding their biological roles and corresponding cellular mechanisms. This challenge in the characterization of variable identities is not only likely to induce biases in interpretability but can also lead to uninterpretable results [56].

Secondly, distinct omics datasets have their own limitations and require complex analysis pipelines prior to performing data integration. For instance, analysis of methylation data is complicated by the uneven distribution of methylation target sequences across the genome requiring specific normalization and scaling strategies [57]. Each omics platform faces unique challenges such as experimental and inherent biological noise, differences among experimental platforms and detection bias [58]. In a similar vein to processing genomic data, a supplementary step is critical to ensure interpretability in metabolomics data: metabolite identification. In an agnostic approach, where metabolites are putatively annotated, integrative analysis can be performed regardless of the metabolite identification step. However in a more specific approach, integrative analysis needs to be performed with regards to whether or not metabolite identification has been realized beforehand. For example, if metabolites have not been identified, data integration would be rather limited to almost purely statistical analysis i.e., classification purposes, prediction purposes or inference of significant variables whereas when metabolite identities are known enrichment analysis methods can be applied. Additional challenges arise since there is often not a one to one relationship between genes and metabolites.

Thirdly, the metabolome is intrinsically different between individual samples due to its rich chemical diversity and hence some observed patterns in metabolic profiles might not be the result of perturbations in the biosystem or phenotype of interest. The integration of an additional omics data can empower analysis and help alleviate these individual variations. For example, Bylesjö et al. [1] used the genomic variation present in the genomic dataset to harness the inter-individual heterogeneity resulting from baseline fluctuation and differentiate it from treatment induced variation. However, integrative analysis sometime needs robust models to account for individual variations [53,59].

Fourthly, metabolomic datasets are characterized by high correlation structures in that many peaks can arise from the same metabolites and metabolites operate within networks of chemical reactions. Hence, two highly correlated metabolites might not be directly related but high correlation can be the result of complex interactions with other metabolites in common.

Finally, additional fundamental challenges are similar to typical challenges that usually arise in data integration frameworks, for example, incompleteness of each data type (i.e., missing values), high dimensionality and heterogeneity of data obtained from multiple sources. It is noteworthy to acknowledge that all data analysis steps need to be performed in account of the technical and experimental limitations of each omics platform including metabolomics. Metabolomics requires relatively high-cost instrumentation, complex data analysis and still suffers from issues of variable sensitivity, high volatility and sample-to-sample variability [60]. As a result, reproducibility is one of the significant hurdles in metabolomics [61]. In fact, the delicate stability of metabolites implies that biological samples need to be handled quickly and uniformly. Minor changes in the experimental conditions or procedure (e.g., different laboratories, external conditions…) can cause critical changes in the observed metabolome which might impact results. Hence, statistical data integration approaches should be appropriately selected based on study design among other criteria we will discuss in the following sections.

## 3. Study Design

Study design including sample and data collection needs to be selected in conjunction with the study research questions and hypotheses (see Section 5). Several scenarios need to be considered with respect to the experimental design such as: How many omics layers will be analysed? How samples will be collected? Which types of omics platforms potentially provide more insight? Is there an intervention effect and is the effect evaluated in different groups e.g., cases vs controls, or is there no intervention, treatment, or exposure administered to participants e.g., cohort study? Are measurements recorded on intervention only, or before and after intervention or at multiple time points? Are measurements from separate omics recorded simultaneously or at lagged time points?

The number of omics layers considered will inherently affect the subsequent analysis steps. In some cases it is sufficient to collect data from two omics layers. For more complex questions the availability of additional omics might empower pattern detection in the integrated data sets [62]. It is important to recognize the biological complexity of organisms when considering study design for multi-omics analysis both in terms of intra-omics and inter-omics variation but also the variation timescale itself. Given the different time scales at which the omics layers operate, the lifetime expression and response time of biomolecules within and between single omics layers differs significantly raising the question of whether observations should be obtained at the same point in time or at different points in time. Currently, no optimal time scale has been suggested by the literature and measurements are often recorded at the same time. However, Canzler et al. [63] proposed to tackle the issue by either generating dense time series to capture as accurate temporal behavior as possible or sampling only at reasonable times before and after the exposure. In this case, practitioners will need to consider a time period that responds to the study focus and objectives e.g., the time period can be longer than the time scale of instant exposure effects if the study is looking at the steady state of metabolic changes. The former option involves denser time series which gives better insights on the dynamic response of a multi-omics system necessitating, nonetheless, higher experimental costs, an increased number of biological samples, and use of potentially more complex computationally intensive statistical models.

Metabolomics data acquisition and metabolic readout highly depends on the choice of the metabolomics platform and whether the type of analysis is targeted or untargeted. Typical experimental platforms for data acquisition purposes use analytical techniques such as 1H nuclear magnetic resonance spectroscopy (NMR) [64,65] and mass spectrometry (MS) [66] to generate appropriate spectral metabolomic profiles of the studied biological system. Appraised for its reproducibility, NMR is advantageous in that it needs minimal sample preparation, is non-destructive and offers high throughput. However, NMR procures a poor sensitivity compared to that of MS which allows signal detection of a larger number of metabolites. MS methods are more popular as they offer higher sensitivity with relatively cheaper instruments despite its high variability due to a greater number of experimental variables including sample preparation procedures, chromatographic separation and ion suppression effects. MS and NMR often offer good agreement between metabolites. Nevertheless, cases of low correlation between metabolic features measured by different platforms may be indicative that the various techniques are detecting different metabolites [67,68]. For instance, Raffler et al. [69] used NMR to find evidence of genotype-metabotype association related to genetic variations in CPS1 locus and found that the strength of the association observed with NMR data is weaker than the association observed with MS. It is important, hence, to carefully consider the platform and type of analysis that most likely generates data exhibiting the strongest association with genetic variation when designing multi-omics studies. Optimally, whenever possible, both NMR and MS (targeted or untargeted) can be used to form a multi-platform approach by combining their respective merits in order to boost metabolite coverage and propose an enhanced readout [70,71,72].

Sample collection is another main issue that needs to be considered when designing multi-omics experiments. Cavill et al. [38] have identified four types of sample collection that should be considered when performing data integration: *repeated study*, *replicate matched study*, *split sample study* and *source matched study*. Briefly speaking, in a repeated study, one omics dataset is first generated following a specific experimental protocol. A second dataset is then obtained by repeating the same experimental protocol usually on a different time or lab. This study design is likely to introduce batch effects into the integrative analysis and is usually complicated to correct for. In certain studies, separate samples from different biological replicates (in the same experiment) are needed to generate metabolomics and transcriptomics data depending on the experimental extraction protocol. This is referred to as replicate matched study. The split sample study is subject to less variation than the repeated or replicate matched study. As its name suggests, it consists of splitting the same biological sample e.g., tissue or biofluid into two samples profiled with different omics technologies. The last case is the source matched study where different fractions of the biological system are used for different analyses for example urine, plasma or faeces. Ideally, samples would be collected from the same biological source for omics platforms. For example Yusufi et al. [73], Gulston et al. [74], Kaluarachchi et al. [75] advantageously used either the source matched study design or the split sample study design to benefit from reduced batch effects.

Ultimately, the experimental design should be informed by the data analysis to limit confounding and batch effects which could be introduced during preparation and storing. Although technical artifacts cannot be completely removed, they can be significantly reduced via a randomized study. It is still important however to recognize these limitations as early as possible in the sample and data collection process and acknowledge their aftermaths.

## 4. Data Types

**Figure 1:**
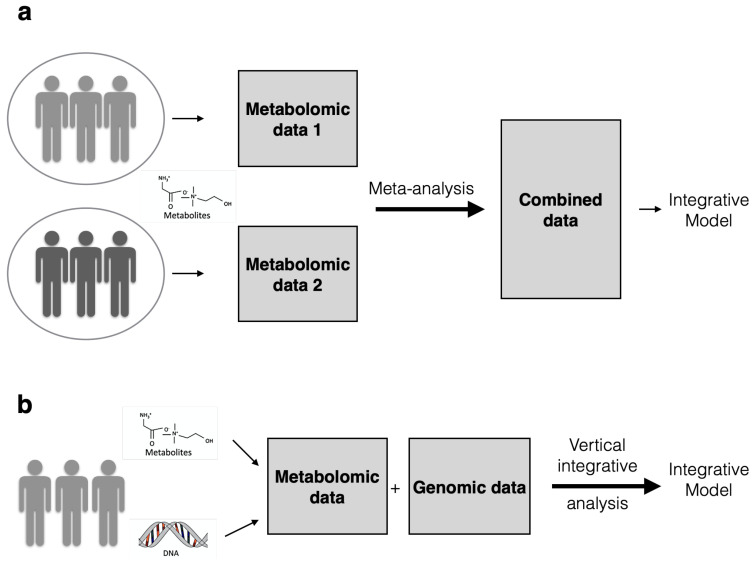
This figure illustrates how different data types can be coupled to each other Example (**a**): Meta-analysis or horizontal integrative analysis involves data collection under different conditions resulting in two datasets that share the same features (e.g., only metabolomic features) but different samples. These observations can be combined into one data matrix after meta-analysis. Example (**b**): In heterogeneous or vertical integrative analysis data are acquired from samples profiled under the same conditions, but do not share the same features e.g., genomic features vs metabolomic features. Strategies that can be used for these types of integrative analysis are depicted in Figure 3.

Generally speaking, the research community is following two kinds of integrative analysis: *horizontal* or *homogeneous meta-analysis* versus *vertical* or *heterogeneous multi-omics analysis* [32,33,40]. Horizontal analysis concerns integration of data where similar entities are measured across different studies, cohorts or labs. On the contrary, vertical analysis deals specifically with different entities such as metabolites and genes measured on the same set of samples (See Figure 1). Homogeneous meta-analysis is subject to a wider range of approaches that could be used for integrative analysis. These statistical approaches, being heavily dependent on a study design involving repeated measurements across different labs or cohorts, are inappropriate for vertical integration purposes. Similarly, various statistical vertical integration techniques cannot be applied for horizontal integrative analysis. The following material of this review aims for a high level overview of data integration techniques that are relevant for consideration in both vertical and horizontal data integration. However, for further reading on methods particularly tailored to each of these approaches we refer the reader to Tseng et al. [34], O’Shea and Misra [35], Toro-Domínguez et al. [76].

## 5. Hypothesis

Ritchie et al. [77] defines multi-omic data integration as “the incorporation of multi-omic information in a *meaningful* way to provide a more comprehensive analysis of a biological point of interest”. Hence, data integration in omics does not only concern data concatenation, linking, coupling or correlation but most importantly the biological consistency of the combined information. Biological consistency is hence a major driver in integrative analysis. For instance, biological consistency is of crucial importance if the integrative analysis method adopted is conceptual, i.e., based on conclusions mostly synthesized by the researcher or the method is model-based, i.e., the biological system can be justly mathematically described, to ensure biological model assumptions are valid [78].

**Figure 2:**
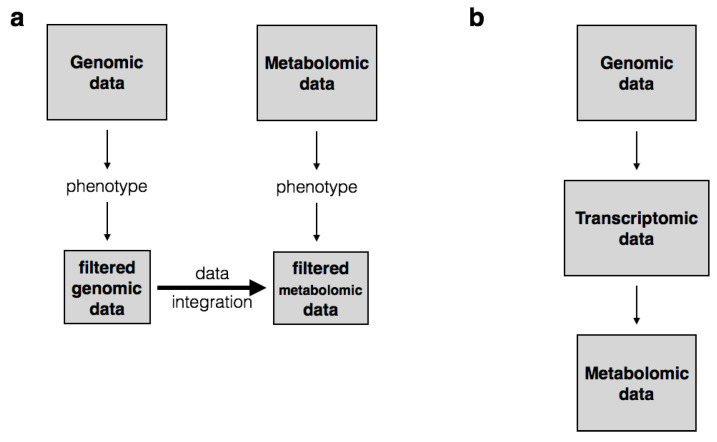
Examples of a multi-stage integrative analysis approach. Example (**a**) illustrates a three-step framework where genomic and metabolomic datasets are concurrently tested for association with the phenotype resulting in smaller datasets. These datasets are then investigated to infer linked variables. Example (**b**) illustrates a typical scenario where genomic variables are tested for association with transcripts which are in turn associated with metabotypes. These metabotypes might for instance explain the expression of a given phenotype. These models are useful for vertical data integration but not suitable for meta-analysis since they assume that different omics entities are observed.

To ensure biological consistency, researchers should question their hypothesis at early stages prior to integrative analysis. In the context of multi-omics integration, one of the main biological hypotheses to think about is whether variation between omics (inter-omics variation) is unidirectional or multi-directional. For instance, if variation is assumed to be unidirectional that is hierarchical from the genome to the metabolome, a multi-staged integrative analysis should be privileged [77]. Multi-staged analysis stands for the process of combining data in consecutive steps where, for example, genomic variables are first associated with transcriptomic variables. Significant transcriptomic variables are then associated with metabotypes (commonly known as metabolic phenotypes or metabolic characteristics [79]). An additional example is where genomic and metabolomic data are separately filtered and associated with a specific phenotype e.g., via GWAS (Genome-Wide Association Studies) and MWAS (Metabolome-Wide Association Studies). The resulting datasets are then tested for mutual association e.g., via metabolome GWAS [7,15,22,41]. This approach is generally carried out to identify changes in phenotypic traits that are induced by changes in the metabolome which in turn are caused by variation in the genome (Figure 2). On the contrary, meta-dimensional analysis supports the hypothesis of simultaneous variation in the genome, transcriptome, proteome and metabolome leading to the phenotype. In other words, the meta-dimensional approach assumes that it is the combination of multiple variables from various data types that results in the phenotype [42,43,52]. In this case, concatenation-based or transformation-based statistical methods can be used to analyze the data simultaneously (See Section 6).

## 6. Data Integration Strategies

**Figure 3:**
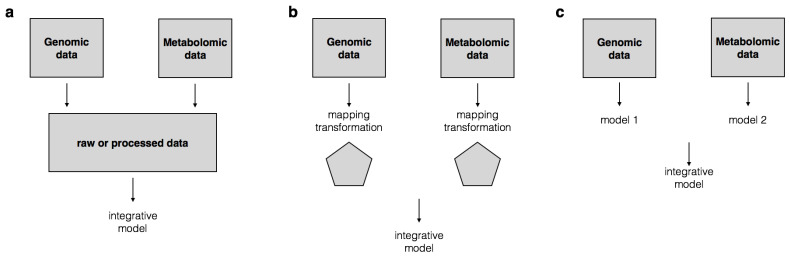
Different data integration strategies. (**a**) illustrates early integration where data is combined into a single data matrix before modeling. (**b**) depicts the intermediate data integration level where data matrices are transformed or mapped into a common meaningful representation before modeling. In (**c**), each data model is generated separately and is then combined with models based on other data types to generate the integrative or high-level model. Early integration is often used in meta-analysis [76]. Intermediate and late data integration strategies can be applied for meta-analysis but such applications are scarce in the literature.

Integrative analysis can also be broadly categorized with respect to different strategies: *low level* or *early integration*, *intermediate integration* and *high level* or *late integration* (Figure 3). In early integration, all inputs are concatenated at raw or processed level to form a single dataset, gathering all the provided information with minimal loss. Hence, one major challenge in early integration is to use an appropriate common representation for datasets from different scales [45,47]. Nonetheless, early integration benefits from two main advantages. First, statistical methods as used for a single data can be applied with slight modifications to the obtained data matrix. Second, it usually preserves information of interaction between omics variables of the input datasets. However, this strategy very much depends on the statistical method that is being applied. For example, PLS-DA, a popular approach in metabolomics for analysis of continuous variables, is not directly applicable to most genomics or microbiome data sets. This strategy is also subject to increasing the high dimensionality of the data by concatenating the total number of variables from all input data into a single one. Hence, dimensionality reduction techniques might be required before performing early integration.

Whereas in early integration transformation shouldn’t change the nature of the data, intermediate integration deals with finding a suitable mapping into another format prior to data combination. This approach covers models that make use of kernel functions or network representation of the data. Kernels have been widely used to capture and transform implicit patterns into explicit schemes by embedding data items into feature space [42,47,80]. By contrast to their superior predictive accuracy, a major disadvantage of kernel-based methods is that they are often difficult to interpret. On the other hand, network based methods are popular in omics data integration as they offer easy integration (by merging edges for example) and enhanced interpretability [48,81]. In metabolomics, ease of interpretability is a major concern and ensures high functionality of the model. Ease of interpretability is, thus, one of the key aspects to consider when developing integrative models.

In late integration, each data type is modeled independently then, the resulting models are used to build an integrative or high level model. In the metabolomics literature, latent variable models, namely principal component analysis (PCA) and partial least squares (PLS) variants, are very popular. They can be used for integrative analysis according to the following procedure [82]: Separate models are fitted for each dataset and score matrices are extracted. These score matrices are then concatenated and used as input for an additional model. The latter is termed as high level model. The main limitation of late integration lies in the fact that information about mutual interactions between different data might be lost as the models are first fitted separately [49,83].

## 7. Study Focus

It goes without saying that the integrative analysis process is greatly influenced by the primary statistical or biological focus of the study. Three non-exhaustive non-exclusive categories of integrative analysis types according to study focus have been identified by Daemen et al. [44], Wang et al. [84]: *sequential integration, biological analysis* and *model-based analysis*. The first category, *sequential integration*, attempts to uncover the development of a phenotype e.g disease as opposed to its prediction. This category mainly answers questions on how does data fusion deepen our understanding of the disease? Does the additional data type confirm the findings of the first data type? Does the additional data type enhance our understanding of the first data type? Such analyses were conducted by e.g., Kleemann et al. [85], Santos et al. [86], Verhoeckx et al. [87]. In general, the authors firstly identify genes that are associated with external perturbations or disease. Secondly, genes are linked to metabolites and corresponding enriched pathways. Metabolomics is a highly suitable source for deriving phenotype biomarkers as well as cross-omics biomarkers since it integrates genetic as well as non-genetic factors. Regression is one of the elementary methods used for cross-omics biomarker discovery in sequential analysis. In a similar fashion to GWAS and MWAS, metabolome GWAS was widely applied to integrate of metabolomic and genomic data but is criticized as analysis is performed in a univariate way. Due the correlation structures inherent to omics data, multivariate regression can be achieved by introducing penalty terms in the frequentist setting or shrinkage priors in the Bayesian setting. Yet, these methods ignore dependence between metabolites in favor of genotype-metabotype dependence. Hence, one of the fundamental challenges that arise in this class of models is to simultaneously model metabolite-metabolite associations and metabolite associations with other omics entities. Biological entities are complex by nature and are arguably regulated by sequences of actions and complex interactions. In this sense, modeling a sequence of observations naturally regulated by chemical processes has proven successful in computational biology [12].

Sequential analysis also comprises disease subtype discovery, commonly achieved via clustering approaches [39,46,50,88,89]. For instance, Le Van et al. [46] propose a clustering model that simultaneously identifies features related to each subtype. In this approach data is integrated via ranked transformation. Clustering for functional analysis was explored by Manikandan et al. [90], Becker et al. [91], Yi et al. [92] in the Bayesian parametric setting and proven to provide more understanding of the forces underlying cellular processes and an unbiased method for researchers to identify related functional clusters. In the Bayesian nonparametric setting more flexible models were implemented by Kirk et al. [29], Yuan et al. [39], Savage et al. [93] via hierarchical models where the notion of “fusion” state was introduced. Hierarchical models offer more degrees of freedom than one-level models and thus allows defining for each data its own parameters that might (or not) be shared. Nonetheless, these models are only applicable for homogeneous integrative analysis, i.e., features that represent the same omics entity (e.g., copy number and expression data). One of the fundamental challenges in this context, hence, lies in the heterogeneity of multiple data types. In [39], the designed model, termed as PSDF, allows clustering of different types of discrete genomic data to identify cancer subtypes, feature selection and infer whether patients exhibit similar profiles across data types. The PSDF model makes use of the Dirichlet process to infer probabilistic cluster assignments and Bayesian hierarchical modeling to integrate genomic data. As it uses discrete data with similar scales, initial data transformation is not required for the PSDF model; however, typically data transformation needs to be realized before applying integrative clustering models.

An important literature body in multi-omics analysis involves the two additional types of integrative analysis based on study focus: *biological integration* and *model-based integration*. In model-based integration, researchers are faced with a range of statistical questions such as which omics variables are associated with the phenotypic changes? Which groups of variables from the different datasets are interacting? Does data fusion improve predictive accuracy of phenotype, disease, temporal behaviour? Is information expressed by the different data types redundant? In this context, an important range of statistical and machine learning methods have been developed in the literature. By way of illustration, kernel-based approaches where proposed to integrate multi-platform metabolomics data such as NMR and GC-MS [42] and multi-platform genomics data [44]. Both authors show that predictive ability of the integrative model significantly outperforms predictive ability of models based on a single type of data. Žitnik and Zupan [83] used matrix factorization to integrate 11 data types to predict gene function in D.discoideum and similarly shows that the integrative model significantly improves prediction compared to single models and is more robust to technical and methodological biases. Metabolomics data are characterized by a high number of metabolic profiles compared to the number of biological samples. Moreover, metabolomic variables are also regulated by complex and strong correlation patterns. Henceforth, dimensionality reduction techniques are of fundamental importance in chemometrics for ease of visualization and interpretation. Dimensionality reduction techniques such as canonical correlation analysis (CCA), principal component analysis (PCA) and partial least squares (PLS)-derived techniques usually involve maximizing a covariance function under orthogonality constraints. In particular, Witten and Tibshirani [43] developed a supervised sparse canonical correlation model in order to find significant linear combinations between copy number and gene expression data. For an extensive reading on the extended family of PCA and PLS methods we refer the reader to Mishra et al. [28], O’Shea and Misra [35], Gromski et al. [94], Mendez et al. [95].

Although CCA-, PCA-, and PLS-derived methods offer rich interpretation in terms of shared and orthogonal components, it is not straight-forward to quantify associations between the different variables and thus limits interpretation with regards to variables’ mutual interrelationships. In biological integration one might use available prior knowledge such as metabolic pathways to reinforce interpretability in dimensionality reduction techniques. The aim of biological integration is to uncover the biological mechanisms of interaction between heterogeneous variables including metabolic pathways, regulatory mechanisms and signaling mechanisms [51,52,54]. In this fashion, the same statistical tool could be used to fulfill different study objectives. For example, Safo et al. [53] built on Witten and Tibshirani [43] to develop a sparse canonical correlation analysis (CCA) model to uncover hidden association patterns between heterogenous data where sparsity is adjusted based on structural information of biological networks. Inherently, the combination of CCA with biological knowledge allows infering underlying biological mechanisms as opposed to model-based integration which only seeks statistically significant associations. It is important to note as well that sequential analysis and biological analysis are fundamentally different. Sequential analysis involves a process where one data is analyzed then a second one is used to confirm or deepen results from the first analysis (the emphasis is not specifically about cellular mechanisms) whereas the focus on biological integration is directly related to underlying cellular mechanisms. Table 3 provides a brief summary of some of the popular and recent tools that support multi-omics analysis, indicating which integrative analysis type based on study focus each tool is most suitable for. For comprehensive surveys on available software for integrative analysis we refer the reader to Mishra et al. [28], Pinu et al. [96].

## 8. Discussion

To conclude, different multi-omics integration approaches can be further classified according to multiple dimensions. Broadly speaking, *data types* and *study design* are parts of the experimental dimension whereas the *strategy* types are parts of the methodological dimension. Finally, *study focus* and underlying variation *hypothesis* reflect the biological dimension regardless of the adopted statistical method. Table 1 summarizes different data integration classes depending on *hypothesis*, *data types*, *strategy*, *study design* and most importantly *study focus*. We also acknowledge that there are three types of multi-omics data integration as identified by Ebbels and Cavill [78]: conceptual, statistical and model-based where the involvement of mathematical procedures in integrative analysis is different.

The study focus is of crucial importance to performing *meaningful* and efficient integrative analysis. Metabolomics is a highly suitable source for deriving biomarkers under this framework as it is the closest layer of the omics cascade that is related to the phenotype. In fact, metabolic profiling is widely used to study genotype-metabotype interactions or metabotype-phenotype interactions such as disease-relevant phenotypes or external stimuli. Identifying interactions between omics variables either in terms of significant statistical associations, biomarker discovery or biological networks enhances data interpretability and represents the end goal of many studies. For the sake of interpretability, an arsenal of mathematical and computational techniques has been developed to achieve such analyses. These techniques include, amongst others, correlation analysis [43,53], integrative regression models [15,22,41] and Bayesian integrative clustering of gene profiles [29,39,93].

It is worth noting that a preliminary examination of the literature at the time of research shed light on two substantial shortcomings. Most of the current integrative analysis approaches are conducted separately from the main stream of the analysis, i.e., as a supplementary step. These two-step integrative approaches are very informative to prioritize data signals, nevertheless, they are not optimal. The heterogeneity of biosystems suggests that interrelationships between the various omics entities is key to exhibiting specific phenotype implying that data integration plays an important role into deciphering mechanisms of biological functions in living organisms [110]. As a consequence, integrative analysis should be part of the main analysis pipeline. On top of that, a close survey of the literature reveals that applications of probabilistic models for integrative analysis in metabolomics are very scarce. This is mostly ascribable to the limited number of available software on probabilistic models in the field which restricted their popularity.

## Figures and Tables

**Table 1:** Classification of different data integration approaches. The examples list is by no means exhaustive.

Integrative Analysis	Description	Examples
*study design*		
Repeated study	In a repeated study the experiment is repeated in another time or place to generate a second type of data.	Cavill et al. [38]
Replicate matched study	In a replicate matched study, biological replicates are used to generate additional types of data.	Cavill et al. [38]
Split sample study	In a split sample study, the same biological sample is split for profiling with different omics technologies.	Cavill et al. [38]
Source matched study	In a source matched study, different samples from the same biological organism are extracted and used to generate different types of data.	Cavill et al. [38]
*data types*		
Horizontal or homogeneous data integration (meta-analysis)	Horizontal integration involves combining measurements of the same omics entities across various cohorts, labs or studies.	Richardson et al. [32], Yuan et al. [39]
Vertical or heterogeneous data integration	Vertical integration involves combining entities from different omics levels, often measured using different platforms.	Richardson et al. [32], Evangelou and Ioannidis [40]
*hypothesis*		
Multi-staged	In multi-staged integration, inter-omics variation (variation between omics) is assumed to be unidirectional from the genome to the metabolome	Nicholson et al. [15], Gieger et al. [22], Krumsiek et al. [41]
Meta-dimensional	In meta-dimensional integration, inter-omics variation is assumed to be multi-directional or simultaneous.	Smolinska et al. [42], Witten and Tibshirani [43], Daemen et al. [44]
*strategy*		
Early integration	Early integration combines two datasets by simply concatenating them into one data.	Fridley et al. [45], Le Van et al. [46]
Intermediate integration	Intermediate integration involves a data transformation step to be performed prior to modeling.	Le et al. [31], Smolinska et al. [42], Lanckriet et al. [47], Guo et al. [48]
Late integration	Late integration consists of combining single data models into a high level model.	Acharjee et al. [49]
*study focus*	*Depending on the study focus, integrative analysis seeks to answer the following:*	
Sequential analysis	Does the additional data type enhance understanding of the first data type?	Yuan et al. [39], Le Van et al. [46], Shen et al. [50]
Biological analysis	What are the underlying processes leading to phenotypical changes? Which mechanisms explain the prevalence of a phenotype?	Hirai et al. [51], Cavill et al. [52], Safo et al. [53], Hong et al. [54]
Model-based analysis	Which variables are phenotypically relevant? significantly associated? Can predictive ability be improved?	Smolinska et al. [42], Witten and Tibshirani [43], Daemen et al. [44]

**Table 2:** Case study examples underlining considerations that researchers should make when carrying out multi-omics experiments and analyses. Integrative analysis that is driven by a hypothesis should result in a data interpretation that links back to that hypothesis (see Section 5). Hence the underlying hypothesis should be considered along with the research question but also at the data interpretation step.

Workflow	Considerations	Choices and Comments
Example from Le et al. [31]		
Study focus	Research questions	Is it possible to predict metabolite abundance from bacteria abundance in inflammatory bowel disease (IBD)? Can we learn the synergistic relationship between the gut microbiome and their surrounding metabolites? These questions suggest an interest in complex associations between the metabolome and the microbiome which will be investigated through model-based analysis. The choice of a model-based analysis highly affects the integrative strategy while requiring it to comply with the hypothesis.
	Hypothesis	As suggested by the research question, the authors assume that there exists intermediate factors that act in the middle of the process that transforms microbes to metabolites and that the processes in which microbes affect metabolites are highly interdependent following a multi-staged integrative approach.
Study design, sample collection and data acquisition	Study type	Paired data from a cohort of inflammatory bowel disease patients.
	Omics layers	Microbiome and metabolome
	Biological samples	Fecal samples
	Platforms	Next-Generation Sequencing (NGS) and LC-MS
	Preprocessing	In addition to the standard pre-processing workflow applied to each platform, the authors used compositional methods e.g., centered log-ratio transformation, to ensure that their workflow will generalize to any pair of omics data.
	Data types	Vertical data integration on paired data with heterogeneous features: microbe abundance and metabolite abundance.
Data analysis	Strategies	Intermediate integration via neural encoder-decoder networks. Non negative weights are imposed on the networks to enforce a unidirectional variation from the microbiome to the metabolome.
Data interpretation	Hypothesis	Microbe abundance is able to reliably predict abundance of a range of metabolites while empowering clinically relevant relationships. The findings also suggest that the “microbe-metabolite axis itself, not just the microbes and metabolites alone, is an IBD-specific biomarker signature.”
Example from Nicholson et al. [15]		
Study focus	Research question	Are there 1H NMR-detectable metabolites in urine or plasma that are strongly influenced by common single-locus genetic variation? This question involves, but not restricted to, a model-based integrative analysis and will guide the study design, data analysis and data interpretation.
	Hypothesis	Variation is unidirectional downstream from genes to metabolites.
Study design, sample collection and data acquisition	Study type	Cohort study
	Omics layers	Genome and metabolome
	Biological samples	Whole-blood, plasma and urine
	Platforms	Untargeted 1H NMR and targeted flow-injection tandem MS: The sets of metabolites observed from the two platforms were minimally overlapping and therefore complementary. The genotyping assay used Illumina arrays.
	Longitudinal profiling	Measurements of heterogeneous omics entities were recorded at the same time point. The longitudinal design allowed detailed variance-components analysis of the sources of population variation in metabolite levels.
	Preprocessing	Preprocessing including metabolite annotation was performed using standard pipelines for each platform.
	Data types	The authors considered two cohorts from the MolPAGE study with the aim of using one cohort to replicate findings of the other one (Sequential integration). Vertical data integration has been performed on Genome-wide SNP genotypes and metabolic features.
Data analysis	Strategies	Early integration through Genome-Wide Metabolic QTL Analysis to identify associations.
Data interpretation	Hypothesis	The mQTLs explained a significant biological population variation in the corresponding metabolites’ concentrations which is well aligned with the hypothesis of a multi-staged integrative analysis. This is also coherent with the research question (study focus) and strategy adopted.

**Table 3:** Brief overview of some multi-omics tools and techniques supporting integrative analysis in alphabetical order.

Resource	Core Integrative Analysis Tasks	Interface	Study Focus	Reference
GAIT-GM	Annotation, network modeling and pathway analysis	Python	Sequential analysis & Biological-based integration	McIntyre et al. [97]
iOmicsPASS	Network-based analysis and predictive feature selection	C++	Model-based integration & Biological-based integration	Koh et al. [98]
INDEED	Network analysis	R	Model-based integration	Zuo et al. [99]
OmicsTIDE	Clustering and visualisation	online	Model-based integration & Sequential integration	Harbig et al. [100]
mbpls	Dimension reduction (Multi-block PLS)	Python	Model-based analysis & Sequential integration	Baum and Vermue [101]
MetaboAnalyst	Enrichment analysis	online, R	Biological-based integration	Xia et al. [102]
MetaBridge	Pathway mapping	online	Biological-based integration	Hinshaw et al. [103]
MetExplore	Pathway mapping and graph-based analysis	online	Biological-based integration	Cottret et al. [104]
mixOmics	Dimension reduction and feature selection	R	Model-based integration	Rohart et al. [105]
multiGSEA	Enrichment analysis	R	Biological-based integration	Canzler et al. [63]
NetMet	Network modeling	online	Biological-based integration	Tal et al. [106]
paintOmics 3	Pathway visualisation	online	Biological-based integration	García-Alcalde et al. [107], Hernández-de Diego et al. [108]
ROSA	Dimension reduction (Multi-block PLS)	R	Model-based analysis & Sequential integration	Liland et al. [109]

## Data Availability

Not applicable.
